# Comprehensive lipidomic analysis of the lipids extracted from freshwater fish bones and crustacean shells

**DOI:** 10.1002/fsn3.2698

**Published:** 2022-01-14

**Authors:** Shuang Lv, Suya Xie, Yunxia Liang, Long Xu, Liangbin Hu, Hongbo Li, Haizhen Mo

**Affiliations:** ^1^ School of Food and Biological Engineering Shaanxi University of Science and Technology Xi'an China; ^2^ Shaanxi Agricultural Products Processing Technology Research Institute Xi'an China; ^3^ College of Food Science and Technology Henan Agricultural University Zhengzhou China

**Keywords:** acylglycerol, aquatic processing by‐products, fatty acids, lipidomic analysis

## Abstract

A comprehensive lipidomic analysis of the lipids extracted from grass carp bones, black carp bones, shrimp shells, and crab shells was performed in this study. First, HPLC analysis revealed that the lipids extracted from shrimp and crab shells contained 60.65% and 77.25% of diacylglycerols, respectively. Second, GC‐MS analysis identified 18 fatty acid species in the lipids extracted from fish bones and crustacean shells, in which polyunsaturated fatty acids (PUFAs) were highly enriched. PUFAs were present at 45.43% in the lipids extracted from shrimp shells. Notably, the lipids extracted from shrimp and crab shells contained a considerable amount of eicosapentaenoic acids and docosahexaenoic acids. Finally, multidimensional mass spectrometry‐based shotgun lipidomics showed that various lipids including acetyl‐L‐carnitine, sphingomyelin (SM), lysophosphatidylcholine, and phosphatidylcholine (PC) were all identified in the lipid samples, but PC and SM were the most abundant. Specifically, the total content of PC in shrimp shells was as high as 6.145 mmol/g. More than 35 species of PC were found in all samples, which were more than other lipids. This study is expected to provide a scientific basis for the application of freshwater fish bones and crustacean shells in food, medicine, and other fields.

## Graphical abstract

The fatty acid composition and lipid molecular species from fish bones and crustacean shells were investigated. The lipids extracted from shrimp and crab shells contained a considerable amount of eicosapentanoic acids and docosahexanoic acids. The total content of PC in shrimp shells was as high as 6.145 mmol/g.

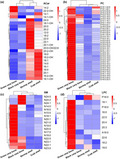

## 1 INTRODUCTION

Fish and crustaceans are widely consumed worldwide as these marine and freshwater products contain abundant functional proteins, lipids, and micronutrients (Byrd et al., 2020; Medeiros et al., 2019). China is one of the leading producers of fish products in the world, with a steady increase observed in fishery products each year. As the blue economy progressed, the fishery product consuming market remarkably expanded. However, massive aquatic processing by‐products such as fish bones and crustacean shells lead to serious environmental issues. The aquatic waste contains high amounts of proteins, polysaccharides, lipids, minerals, and flavor substances (Nawaz et al., 2018). Therefore, the utilization of aquatic waste should receive a lot more attention.

Fish bones are rich in fat, proteins, polysaccharides, minerals, and flavor substances (Nawaz et al., 2018). Different types of calcium supplementation products were made from fish bones, including calcium polypeptide chelate, calcium amino acid chelate, and bone calcium tablets. Fish bones were also used as ingredients for making snack foods and seasonings. Crustacean shells (especially from shrimps and crabs) are abundant in proteins, lipids, fat‐soluble vitamins, phosphorus carbonate, calcium carbonate, and chitin (Hamed 2016). The protein and lipid content in snow crab shells was 34.2% and 17.1%, respectively (Lage‐Yusty et al., 2011). Some beneficial omega‐3 PUFAs, especially EPAs and DHAs, were found in the lipids extracted from fish bones and crustacean shells. These PUFAs are associated with anti‐inflammation and cognition improvement (Bao et al., 2016; Bazinet & Layé, 2014; Wysoczański et al., 2016). It was documented that long‐term consumption of fish oils helped to prevent osteoporosis and skeletal development (Wauquier et al., 2012). Moreover, fish oil waste is a potential source of biofuel (Fu et al., 2017).

Up until now, a great deal of work has been performed investigating lipids extracted from various fish and crustaceans (Chang et al., 2017; Zhang et al., 2021). However, a comprehensive lipidomic analysis of the lipids extracted from fish bones and crustacean shells was lacking. In the study presented here, we focused on the lipids extracted from two types of fish bones (i.e., grass carp and black carp bones) and two types of crustacean shells (i.e., shrimp and crab shells). Even though lipid profiles are affected by the habitats and origins of aquatic animals (Liu et al., 2020), the characterization of the lipids from fish bones and crustacean shells performed in our study was thought to provide fundamental lipidomic data. The analysis of the lipids from fish bones and crustacean shells would contribute to the potential utilization of this aquatic waste.

## 2 MATERIALS AND METHODS

### 2.1 Samples and their preparation

Shrimps, crabs, grass carp, and black carp were purchased from a local wet market in Weiyang District. Fish bones were separated from flesh. Shrimps and crabs were dehulled to obtain their shells. The resulting fish bones, shrimp, and crab shells were freeze‐dried under vacuum overnight. Then, these bones and shells were crushed into powders using a pulverizer (FW‐100D; Taisite Instrument Co., LTD). Samples were placed in zip‐lock bags and stored at −20°C.

### 2.2 Chemicals and standards

Methanol and *n*‐hexane (HPLC grade, ≥99.5%) were purchased from Kemiou Chemical Reagent Co., Ltd.. Chloroform (≥99.7%) was purchased from Sinopharm Chemical Reagent. Concentrated sulfuric acid (98%) was purchased from Thermo Fisher Scientific. Lastly, NaCl (≥99.5%) and butylated hydroxytoluene (≥99.8%) were purchased from Tianli Chemical Reagent.

### 2.3 Analytical methods

#### 2.3.1 Lipid extraction

The lipids from carp bones and crustacean shells were extracted with chloroform/methanol/water (8:4:3, *v*/*v*/*v*) containing 0.005% BHT (sample to solvent ration = 1:15), assisted by ultrasound (40 kHz, 100 W) at 30℃ for 20 min, which was repeated for three times. The combined lipid extracts were filtered and evaporated using a rotary evaporator (RE100‐Pro; Forging Technology Development Co., Ltd.,) at 40℃.

#### 2.3.2 Analysis of acylglycerol species by NP‐HPLC

The analysis of acylglycerol species was performed using a normal‐phase high‐performance liquid chromatography (NP‐HPLC) with a Phenomenex Luna silica gel column (250 × 4.6 mm, 5 µm) at a column temperature of 30°C. N‐hexane/2‐propanol/formic acid (21/1/0.003, v/v/v) was used as the mobile phase, and the flow rate was set at 1 ml/min.

#### 2.3.3 Analysis of fatty acid methyl esters (FAMEs) by GC‐MS

All lipids were methylated to FAMEs as follows: The extracted sample (20 μl) was mixed with 1% sulfuric acid in methanol (1.5 ml) and heated for 1 hr at 80°C in small sealed tubes. Aqueous saturated NaCl solution (1 ml) and demineralized water (1 ml) were added after the mixture was cooled to room temperature. Such generated FAMEs were extracted with *n*‐hexane (2 ml). The organic phase was transferred to a standard glass autosampler vial for GC‐MS analysis.

The analysis of FAMEs was performed on an Agilent 7000D GC‐MS Triple Quad (Agilent Technologies, Inc.) equipped with a DB‐23 column (30 m × 0.25 mm, 0.25 μm). All measurements in the full‐scan mode (*m*/*z* = 50–500) were performed as follows: The initial temperature (130°C) was held for 1 min and then raised to 230°C with a ramp of 5°C/min. The final temperature (230°C) was held for 5 min, resulting in a total run time of 26 min. The injection volume was set at 1 µl, with split injection at a split ratio of 1:20 using an autosampler.

#### 2.3.4 Lipidomic analysis by MDMS‐SL

The lipids extracted from carp bones and crustacean shells (100 mg each) were reconstituted in 200 μl of chloroform/methanol (1/1, v/v; Yang, Cheng et al., 2009). Lipidomic analysis was performed on a triple‐quadrupole mass spectrometer (Thermo TSQ Quantiva,), equipped with an automated nanospray ion source (TriVersa NanoMate, Advion Bioscience Ltd.), and operated using Xcalibur system software (Han et al., 2008). Identification and quantification of different lipid classes and individual species was performed by multi‐dimensional mass spectrometry‐based shotgun lipidomics (MDMS‐SL) as described previously (Yang, Cheng, et al., 2009), based on lipidomic principles (Wang et al., 2016).

## 3 RESULTS

### 3.1 Acylglycerol species

The acylglycerol species in fish bones, shrimp shells, and crab shells determined by NP‐HPLC are presented in Table 1.

**TABLE 1:** Acylglycerol species (wt%) in the lipids extracted from fresh fish bones and crustacean shells

Samples	TAG (%)	DAG (%)		MAG/%	
		1,3‐DAG (%)	1,2‐DAG (%)	1‐MAG (%)	2‐MAG (%)
Grass carp bone	98.98 ± 0.06	0.10 ± 0.01	0.92 ± 0.06	ND	ND
Black carp bone	96.89 ± 0.04	0.56 ± 0.02	2.55 ± 0.04	ND	ND
Shrimp shell	37.13 ± 1.04	8.55 ± 0.51	52.10 ± 1.60	2.22 ± 0.01	ND
Crab shell	22.75 ± 0.79	7.30 ± 0.92	69.95 ± 0.13	ND	ND

Abbreviation: ND, not detected.

Results showed similarities in triacylglyceride (TAG) and 1,3‐DAG content between the lipids extracted from the bones of grass and black carp, as well as between the lipids extracted from shrimp and crab shells. Specifically, TAG content in the lipids from fish bones (96%~99%) was three times higher than what was observed in the lipids from crustacean shells (22%~38%). The content of 1,3‐DAG in the lipids extracted from crustacean shells (7%~9%) was significantly higher than what was observed in the lipids extracted from carp bones (0.1%~0.6%). It is important to note that the lipids extracted from shrimp and crab shells contained 60.65% and 77.25% of DAGs, respectively, where 1,2‐DAG was the most abundant species. 2‐Monoacylglycerol (2‐MAG) was not present in any of these lipids, and only a small amount of 1‐MAG (2.25%) was identified in the lipids extracted from shrimp shells.

### 3.2 Fatty acid profiles

The fatty acid profiles of the lipids from fish bones, shrimp shells, and crab shells are shown in Table 2.

**TABLE 2:** Fatty acid species (mol%) in the lipids extracted from fresh fish bones and crustacean shells

Abbreviation	Fatty acids	Grass carp bone	Black carp bone	Shrimp shell	Crab shell
C14:0	Myristic acid	1.01	0.85	0.62	0.59
C15:0	Pentadecane acid	0.1	0.11	0.48	0.15
C16:0	Palmitic acid	18.73	20.85	20.3	17.22
C17:0	Heptadecanoic acid	0.08	ND	1	0.56
C18:0	Stearic acid	3.73	4.91	8.64	10.44
C20:0	Eicosanoic acid	ND	ND	0.32	0.42
∑SFA		23.65	26.72	31.36	29.38
C16:1 (n−9)	Palmitoleic acid	5.23	3.51	1.53	3.43
C17:1 (n−7)	*cis*−10‐Heptadecenoic acid	0.1	ND	ND	0.37
C18:1 (n−9)	Oleic acid	40.7	40.49	20.93	25.52
C20:1 (n−9)	*cis*−11‐Eicosenoic acid	0.66	0.71	0.83	0.58
∑MUFA		46.69	44.71	23.29	29.9
C18:2 (n−6)	Linoleic acid	22.84	21.81	22.63	7.2
C18:3 (n−6)	γ‐linolenic acid	0.24	0.51	0.25	ND
C18:3 (n−3)	α‐Linolenic acid	1.62	0.86	1.91	0.81
C20:2 (n−6)	*cis*−11,14‐Eicosadienoic acid	0.82	0.38	1.66	0.69
C20:3 (n−6)	*cis*−8,11,14‐Eicosatrienoic acid	0.78	0.24	ND	ND
C20:4 (n−6)	Arachidonic acid	1.39	1.65	4.04	4.78
C20:5 (n−3)	EPA	ND	ND	6.29	12.50
C22:6 (n−3)	DHA	ND	0.78	4.09	9.89
∑PUFA		27.69	26.23	40.87	35.87
EPA + DHA		ND	0.78	10.38	22.39
n−3 PUFAs		1.62	1.64	12.29	23.2
n−6 PUFAs		25.25	24.21	26.92	11.98
n−6 PUFAs/n−3 PUFAs		15.59	14.76	2.19	0.52
Others		1.97	2.34	4.49	4.84

Abbreviations: MUFA, monounsaturated fatty acid; ND, not detected; PUFA, polyunsaturated fatty acid; SFA, saturated fatty acid.

More than 18 fatty acids, including 6 saturated fatty acids (SFAs), 4 monounsaturated fatty acids (MUFAs), and 8 polyunsaturated fatty acids (PUFAs), were identified by GC‐MS in the lipids extracted from carp bones and crustacean shells. These findings highlighted the similarities in fatty acid composition between the lipids from grass carp and black carp bones, as well as between the lipids from shrimp and crab shells. SFAs accounted for 23%~27% and 29%~32%, respectively, in the lipids from these fish bones and crustacean shells, where palmitic acid (C16:0) and stearic acid (C18:0) were the most abundant ones. Oleic acid (C18:1) was the most enriched MUFA in all samples. In addition, small amounts of palmitoleic acid (C16:1) and *cis*‐11‐eicosenoic acid (C20:1) were found in these lipid samples. Fatty acid profiling also showed that linoleic acid (C18:2) was the most abundant PUFA in the lipids from shrimp shells and carp bones, both of which contained similar levels. Notably, the content of linoleic acid in the lipids from crab shells was only 1/3 of what was observed in the shells from shrimp and fish bones. EPA was not found in the lipids from fish bones, and only a low level of DHA (0.78%) was identified in the lipids from black carp bones. This may be because EPA and DHA do not exist or are only present at negligible amounts in freshwater fish (£uczyñska et al., 2014). In contrast, both the lipids from shrimp and crab shells contained EPA and DHA. The total content of EPA (C20:5) and DHA (C22:6) in the lipids from crab shells (22.39%) was more than two times higher than that found in the lipids extracted from shrimp shells (10.38%). Moreover, the levels of arachidonic acid (ARA, C20:4) in the lipids from crustacean shells were almost three times more than those found in the lipids from carp bones. An equal amount of n‐3 PUFAs was found in the lipids from two types of carp bones. The amount of n‐3 PUFAs in the lipids from crab shells was nearly two times more than that found in the lipids extracted from shrimp shells. The n‐6 PUFAs existed in similar amounts (24%~27%) in the lipids from grass carp bones, black carp bones, and shrimp shells. Moreover, the amount of n‐6 PUFAs in the lipids from these three species was two times more than that found in the lipids of crab shells. The ratio of n‐6 PUFAs to n‐3 PUFAs, which can be used as an effective index for the production of high value‐added products, was significantly higher in fish bone lipids compared with crustacean shell lipids.

### 3.3 Lipidomic analysis performed by MDMS‐SL

**FIGURE 1:**
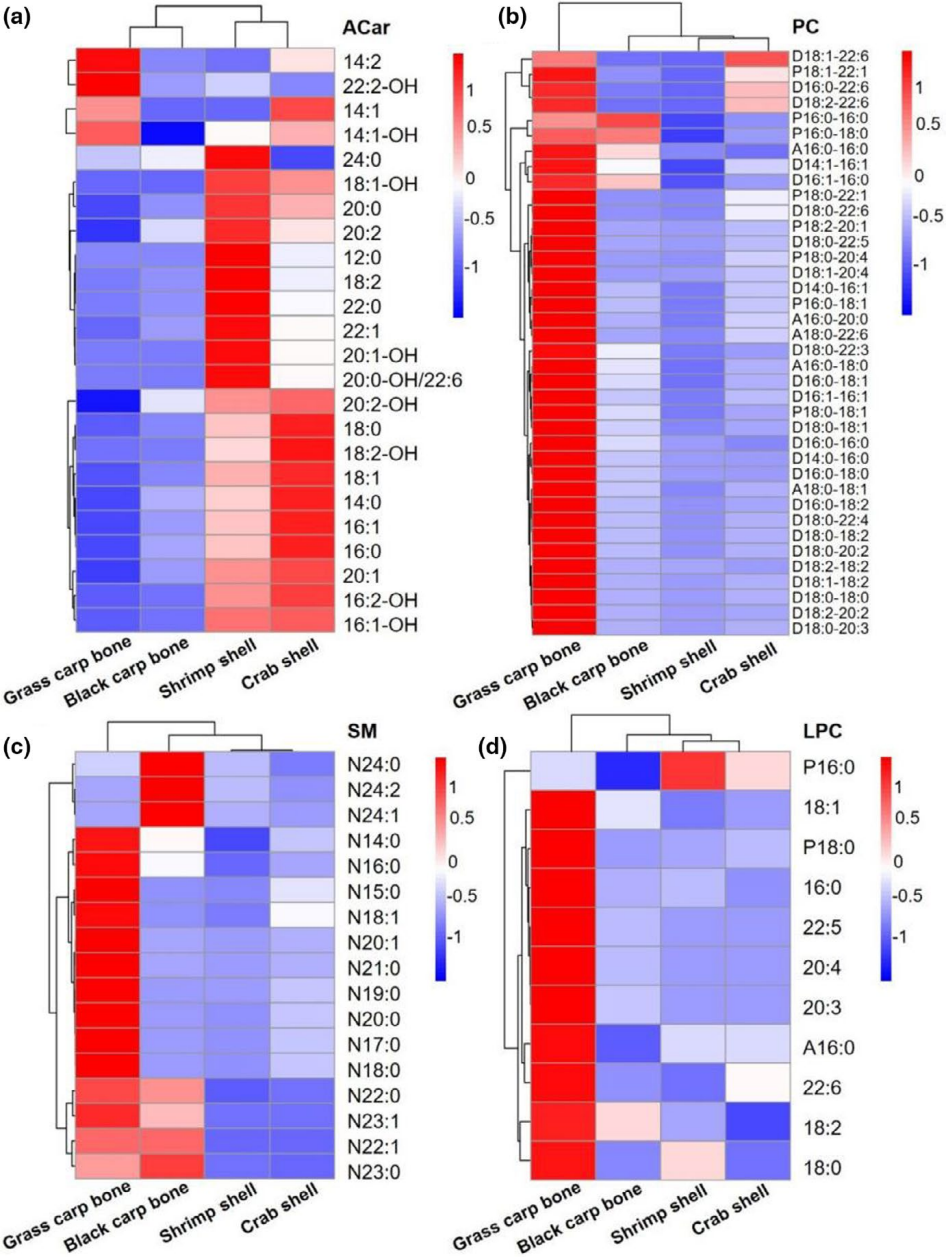
Heat map of the lipidome extracted from fresh fish bones and crustacean shells

The lipidomic analysis from fish bones and crustacean shells was performed using MDMS‐SL. A variety of lipids, including ACar, SM, LPC, and PC, were identified (Tables S1‐S4). Heat maps of the lipidome extracted from these samples are shown in Figure 1.

A total of 24 ACar species were identified in the lipids extracted from crustacean shells, while the lipids from fish bones contained less than 20. The total content of ACar in the lipids from crab shells (14.14 nmol/g) was significantly higher than what was observed in the lipids from fish bones (1.26 nmol/g) and shrimp shells (11.80 nmol/g). Specifically, the most enriched ACar species in the lipids from crab shells were ACar‐16:0 (3.24 nmol/g), ACar‐18:0 (2.73 nmol/g), and ACar‐18:1 (3.60 nmol/g). ACar‐16:0 and ACar‐18:1 were also the most abundant ACar species present in the lipids extracted from blackfish bones and shrimp shells.

With respect to PC, 36 PC species were identified in the lipids from grass carp bones, while 38 PC species were found in other samples. D14:1–16:1 and D18:0–22:3 were not identified in the lipids from grass carp bones. A large amount of PC (6.145 mmol/g) was found in the lipids from shrimp shells, in which the most abundant PC species was D16:0–18:1 (1.487 mmol/g). Only small levels of PC (198.65 nmol/g) were identified in the lipids from grass carp bones in comparison with the lipids from shrimp shells. In addition, similar PC species were found in the lipids from blackfish bones and crab shells.

With respect to SM, 17 SM species were identified in the lipids from fish bones and crustacean shells. The total levels of SM in the lipids from shrimp shells (930.87 nmol/g) were significantly higher than those in the lipids from other species, where N16:0 (205.62 nmol/g) and N18:0 (275.47 nmol/g) were the most abundant species and N24:0 (1.27 nmol/g) contained the lowest levels. Notably, the total amount of SM in the lipids from black carp bones (283.31 nmol/g) was seven times greater than what was observed in the lipids from grass carp bones (37.03 nmol/g). SM in the lipids from shrimp shells was over five times greater than the amount present in the lipids from crab shells. Similarly, SM‐N16:0 was abundant in all lipids.

With respect to LPC, more than 10 LPC species were identified in the lipids from fish bones and crustacean shells. The results showed that the content of LPC in the lipids from fish bones was significantly higher than that in the lipids from crustacean shells. Specifically, LPC in the lipids from grass carp bones (56.72 nmol/g) was over four times greater than the amount observed in black carp bone lipids (13.86 nmol/g), while the level of LPC in the lipids from shrimp shells (11.47 nmol/g) was close to that of the lipids from crab shells (9.81 nmol/g). LPC‐18:1 and LPC‐16:0 were the most abundant LPC species in the lipids from fish bones and crustacean shells. Unexpectedly, LPC‐22:5 content and LPC‐22:6 content in the lipids from grass carp bones were significantly higher than what was observed in the lipids extracted from other species.

## 4 DISCUSSION

In recent years, the marine economy led to the rapid development of an aquatic industry in China, but at the same time resulted in a great deal of aquatic byproduct wastes. In addition to fish bones and crustacean shells, some other examples of aquatic waste include fish skin, heads, and fins, all of which contain proteins, polysaccharides, and other high value materials. Studies have shown that the chitosan in crustacean shells not only serves as an antioxidant and antimicrobial (López‐Pedrouso et al., 2019) but also has good membrane forming abilities. Therefore, chitosan is widely used for food preservation, cosmetics, pharmaceutical industries, and food packaging material (Kizhekkedath et al., 2012). Moreover, materials made of chitosan and calcium phosphate are applied in the biomedical and environmental fields, such as in drug delivery, wound healing, adsorption of organic compounds, and heavy metals from polluted water ( Salama, 2021). Fish waste is rich in collagen that can be used in gelatin production (Kizhekkedath et al., 2012). If aquatic by‐products are utilized, waste will be reduced and the environment will be protected.

Presently, methods used to extract lipids from aquatic products include cooking (Medeiros et al., 2019), solvent extraction (Shahi et al., 2018), supercritical fluid extraction (Kuvendziev et al., 2018), pressing extraction (Haq et al., 2017), solid‐phase extraction (Shen et al., 2015), and enzymatic hydrolysis extraction (Wang et al., 2019). Cooking methods may lead to the loss of heat‐sensitive components in lipids due to high temperatures. Supercritical fluid extraction technology is mainly used to extract components containing high free fatty acids, contains a fast extraction rate, and is nontoxic and solvent‐free, but difficult for large‐scale production due to high costs (Haq et al., 2017). Enzymatic hydrolysis extraction can improve the quality and yield of lipids (Wang et al., 2019), but the large amount of enzyme leads to high cost. The Folch method applying chloroform–methanol as a solvent is the most commonly used method. Rincón‐Cervera et al. (2020). used the Folch method to extract lipids from edible species of fish and shellfish captured in the South Pacific. Sun et al. (Sun et al., 2019) extracted total lipids from two types of Antarctic krills using the Folch method, where the ratio of solvent to sample was improved, saving the cost but without affecting the extraction rate. Moreover, the ultrasonic‐assisted solvent extraction method was adopted to improve lipid extraction efficiency (Zhang et al., 2014).

Goremykina et al. (2016) studied the glyceride composition of the oil from sea buckthorn located in Altai Krai using high‐temperature gas chromatography. NP‐HPLC was used in our study to analyze acylglycerol species in the lipids extracted from fish bones and crustacean shells. The NP‐HPLC analysis showed that the lipids from shrimp and crab shells contained a significant amount of DAGs and a small number of MAGs. It was previously reported that 1,3‐DAG‐rich oil is low in calories, which can decrease serum and liver cholesterol as well as TAG levels and inhibit fat accumulation in vivo after consumption ( Devi et al., 2018; Meng et al., 2004). Therefore, the lipids from crustacean shells containing high DAG content can be used as a new food material to maintain a healthy weight and prevent lifestyle‐related diseases caused by obesity. However, it was suggested that the lipids rich in DAG and MAG content promote the formation of 3‐MCPD esters and GES during oil refining, leading to edible oil contamination (Freudenstein et al., 2013). Thus, additional studies regarding the pros and cons of acylglycerols are needed.

Gas chromatography (GC) is a method used to analyze fatty acid profiles in the lipids from aquatic products. Gonalves et al. (2020) identified the fatty acid composition of 14 marine fish from the northeast coast of Brazil using gas chromatography with a flame ionization detector (GC‐FID). GC‐FID analysis showed that 53 fatty acid species were identified, and the fatty acid composition of different fish varied. Rodrigues et al. (Rodrigues et al., 2020) used GC to analyze the fatty acid profiles of 4 freshwater fish species. All samples showed a pattern where PUFA levels were greater than MUFA levels, which were greater than SFA levels. In addition, the changes during frying on fatty acid composition of Nile tilapia (*Oreochromis niloticus*) fish muscle were analyzed using GC‐MS (Mekonnen et al., 2020). In this study, fatty acid composition in the lipids extracted from fish bones and crustacean shells was identified using GC‐MS. The results showed that 18 fatty acids were identified, in which PUFAs were highly enriched. The total content of EPA and DHA in the lipids from crustacean shells was significantly higher than what was observed in fish bones. Both EPA and DHA are beneficial to human health, especially for the prevention of neurodegenerative diseases (Anderson et al., 2019).

Individual lipid molecular species are identified and quantified directly from lipid extracts of biological samples using MDMS‐SL, a well‐established technology for lipid analysis (Han, 2010). Yang, Zhao et al. (2009)) conducted research for the identification and quantitation of choline‐containing phospholipid molecular species using MDMS‐SL after intrasource separation. In this study, a variety of lipids, including ACar, SM, PC, and LPC, were identified from the lipids extracted from fish bones and crustacean shells. Among these, the most abundant species were PC and SM, both of which are phospholipids and have the ability to improve immunity and enhance organ and tissue functions.

## 5 CONCLUSIONS

This study provided a comprehensive lipidomic analysis of the lipids extracted from freshwater fish bones (i.e., grass carp and black carp bones) and crustacean shells (i.e., shrimp and crab shells). NP‐HPLC analysis showed that the DAG content in the lipids from crustacean shells was greater than the content observed in fish bones. GC‐MS analysis showed that PUFAs were abundant in all lipid samples. Notably, the lipids extracted from shrimp and crab shells contained a large amount of EPA and DHA, which are associated with anti‐inflammation and cognitive improvement. Finally, ACar, PC, SM, and LPC were identified in these samples; PC and SM were rich in these samples. Interestingly, PC, SM, and LPC are the main components of biofilms. Therefore, the utilization of lipid resources from aquatic waste, such as fish bones and crustacean shells, should be considered as a way to protect the environment while improving nutrition.

## CONFLICT OF INTERESTS

The authors declare no conflict of interest.

## AUTHOR CONTRIBUTIONS

**Shuang Lv:** Investigation (lead); Writing – original draft (equal). **Suya Xie:** Data curation (equal); Formal analysis (equal); Software (equal). **Yunxia Liang:** Software (equal); Visualization (equal). **Long Xu:** Data curation (equal); Methodology (lead). **Liangbin Hu:** Formal analysis (equal); Project administration (lead); Resources (equal). **Hongbo Li:** Data curation (lead); Funding acquisition (supporting); Writing – review & editing (lead). **Haizhen Mo:** Funding acquisition (supporting); Supervision (lead).

## ETHICAL STATEMENTS

This study does not involve any human or animal testing.

## Supporting information

Table S1‐S4

## Data Availability

The data that support the findings of this study are available from the corresponding author upon reasonable request.
