# Supplementary material for: Comprehensive lipidomic analysis of the lipids extracted from freshwater fish bones and crustacean shells
Source: Food Sci Nutr. 2022 Jan 14;10(3):723–30. doi: 10.1002/fsn3.2698 (PMC8907742; doi:10.1002/fsn3.2698)
Supplement: Supplementary file 1 — Table S1‐S4 [file FSN3-10-723-s001.doc]

**Comprehensive lipidomic analysis of lipids extracted from freshwater fish bones and crustacean shells**

**^1, 2^Shuang Lv, ^1, 2^Suya Xie, ^1, 2^Yunxia Liang, ^3^Long Xu, ^1, 2^Liangbin Hu, ^1, 2^,*Hongbo Li, and ^1, 2^,*Haizhen Mo^a^**

^1^ School of Food and Biological Engineering, Shaanxi University of Science and Technology, Xi’an 710021, [China;](mailto:China；hongbo715@163.com)

^2^ Shaanxi Agricultural Products Processing Technology Research Institute, Xi’an 710021, [China;](mailto:China；hongbo715@163.com)

^3^ College of Food Science and Technology, Henan Agricultural University, Zhengzhou, 450002, China;

**^*^** Correspondence: hongbo715@163.com；mohz@sust.edu.cn

**Supplemental Tables**

**Table S1** ACar species (nmol/g) in the lipids extracted from fresh fish bones and crustacean shells.

**Table S2** PC species (nmol/g) in the lipids extracted from fresh fish bones and crustacean shells.

**Table S3** SM species (nmol/g) in the lipids extracted from fresh fish bones and crustacean shells.

**Table S4** LPC species (nmol/g) in the lipids extracted from fresh fish bones and crustacean shells.

**Table S1 ACar species (nmol/g) in the lipids extracted from fresh fish bones and crustacean shells.**

| **Species** | **[Acyl-Carnitine + H]^+^** | **Grass carp bone** | **Black carp bone** | **Shrimp shell** | **Crab shell** |
| --- | --- | --- | --- | --- | --- |
| 12:0 | 344.280 | 0.00 | 0.00 | 0.50 | 0.14 |
| 14:2 | 368.280 | 0.27 | 0.02 | 0.01 | 0.12 |
| 14:1 | 370.296 | 0.13 | 0.06 | 0.06 | 0.15 |
| 14:0 | 372.311 | 0.02 | 0.12 | 0.24 | 0.41 |
| 14:1-OH | 386.291 | 0.39 | 0.03 | 0.25 | 0.32 |
| 16:1 | 398.327 | 0.01 | 0.14 | 0.40 | 0.67 |
| 16:0 | 400.343 | 0.05 | 0.75 | 1.91 | 3.24 |
| 16:2-OH | 412.306 | 0.00 | 0.01 | 0.14 | 0.18 |
| 16:1-OH | 414.322 | 0.00 | 0.02 | 0.37 | 0.39 |
| 18:2 | 424.343 | 0.05 | 0.19 | 1.94 | 0.63 |
| 18:1 | 426.358 | 0.01 | 0.53 | 2.30 | 3.60 |
| 18:0 | 428.374 | 0.00 | 0.26 | 1.50 | 2.73 |
| 18:2-OH | 440.338 | 0.00 | 0.01 | 0.11 | 0.23 |
| 18:1-OH | 442.353 | 0.00 | 0.00 | 0.29 | 0.22 |
| 20:2 | 452.374 | 0.00 | 0.07 | 0.18 | 0.10 |
| 20:1 | 454.390 | 0.00 | 0.09 | 0.27 | 0.34 |
| 20:0 | 456.405 | 0.01 | 0.05 | 0.23 | 0.16 |
| 20:2-OH | 468.369 | 0.01 | 0.06 | 0.09 | 0.10 |
| 20:1-OH | 470.385 | 0.00 | 0.00 | 0.11 | 0.04 |
| 20:0-OH/22:6 | 472.400 | 0.00 | 0.00 | 0.30 | 0.11 |
| 22:1 | 482.421 | 0.04 | 0.06 | 0.22 | 0.11 |
| 22:0 | 484.437 | 0.00 | 0.01 | 0.15 | 0.05 |
| 22:2-OH | 496.400 | 0.23 | 0.07 | 0.09 | 0.06 |
| 24:0 | 512.468 | 0.05 | 0.06 | 0.12 | 0.02 |
| Total |  | 1.26 | 2.60 | 11.80 | 14.14 |

**Table S2 PC species (nmol/g) in the lipids extracted from fresh fish bones and crustacean shells.**

| **Species** | **[PC + Li]^+^** | **Grass carp bone** | **Black carp bone** | **Shrimp shell** | **Crab shell** |
| --- | --- | --- | --- | --- | --- |
| D14:1-16:1 | 708.516 | 0 | 0.46 | 1.06 | 0.33 |
| D14:0-16:1 | 710.531 | 0.28 | 2.2 | 11.25 | 2.22 |
| D14:0-16:0 | 712.547 | 1.99 | 6.67 | 46.72 | 1.03 |
| P16:0-16:0 | 724.583 | 9.68 | 64.28 | 52.07 | 20.19 |
| A16:0-16:0 | 726.599 | 5.17 | 17.43 | 33.24 | 3.52 |
| D16:1-16:1 | 736.547 | 0.26 | 8.44 | 40.69 | 6.56 |
| D16:1-16:0 | 738.563 | 3.89 | 97.01 | 160.69 | 35.71 |
| D16:0-16:0 | 740.578 | 18.64 | 75.18 | 319.36 | 7.61 |
| P16:0-18:1 | 750.599 | 2.98 | 21.95 | 119.74 | 25.87 |
| P16:0-18:0 | 752.615 | 9.67 | 73.42 | 80.11 | 27.45 |
| A16:0-18:0 | 754.63 | 1.17 | 11.73 | 37.64 | 7.21 |
| D16:0-18:2 | 764.578 | 8.22 | 60.76 | 421 | 34.73 |
| D16:0-18:1 | 766.594 | 33.69 | 418.43 | 1486.54 | 264.4 |
| D16:0-18:0 | 768.609 | 2.52 | 17.31 | 132.78 | 4.19 |
| P18:0-18:1 | 778.63 | 0.85 | 19.71 | 79.98 | 9.95 |
| A18:0-18:1 | 780.646 | 1.62 | 21.89 | 130.58 | 15.53 |
| A16:0-20:0 | 782.661 | 2.85 | 14.88 | 102.93 | 24.28 |
| D18:2-18:2 | 788.578 | 24.56 | 29.16 | 228.22 | 20.37 |
| D18:1-18:2 | 790.594 | 8.52 | 52.7 | 746.69 | 44.15 |
| D18:0-18:2 | 792.609 | 4 | 42.36 | 366.69 | 33.71 |
| D18:0-18:1 | 794.625 | 7.77 | 90.3 | 440.36 | 43.64 |
| D18:0-18:0 | 796.641 | 0.44 | 1.84 | 28.29 | 1.93 |
| P18:0-20:4 | 800.615 | 1.67 | 2.58 | 64.09 | 11.51 |
| P18:2-20:1 | 802.63 | 0.55 | 2.93 | 85.44 | 10.1 |
| D16:0-22:6 | 812.578 | 8.55 | 17.59 | 220.31 | 135.83 |
| D18:1-20:4 | 814.594 | 18.43 | 18.42 | 213.95 | 43.5 |
| D18:2-20:2 | 816.609 | 7.54 | 15.48 | 115.14 | 11.32 |
| D18:0-20:3 | 818.625 | 1.2 | 8.88 | 94.9 | 6.62 |
| D18:0-20:2 | 820.641 | 0.49 | 3.77 | 30.17 | 2.98 |
| A18:0-22:6 | 826.63 | 0.22 | 1.78 | 20.97 | 4.32 |
| P18:1-22:1 | 832.677 | 0.02 | 0.46 | 4.82 | 2.29 |
| P18:0-22:1 | 834.693 | 0.05 | 0.43 | 10.43 | 2.88 |
| D18:2-22:6 | 836.578 | 0.85 | 1.93 | 38 | 23.07 |
| D18:1-22:6 | 838.594 | 2.53 | 4.33 | 53.88 | 60.84 |
| D18:0-22:6 | 840.609 | 4.59 | 5.82 | 65.81 | 21.12 |
| D18:0-22:5 | 842.625 | 2.77 | 3.43 | 34.05 | 5.48 |
| D18:0-22:4 | 844.641 | 0.42 | 2.23 | 15.8 | 1.83 |
| D18:0-22:3 | 846.656 | 0 | 3.29 | 10.85 | 0.83 |
| Total |  | 198.65 | 1241.46 | 6145.25 | 979.1 |

P, plasmenyl-; A, plasmanyl-.

**Table S3 SM species (nmol/g) in the lipids extracted from fresh fish bones and crustacean shells.**

| **Species** | **[SM + Li]^+^** | **Grass carp bone** | **Black carp bone** | **Shrimp shell** | **Crab shell** |
| --- | --- | --- | --- | --- | --- |
| N14:0 | 681.552 | 0.17 | 36.32 | 81.98 | 25.38 |
| N15:0 | 695.568 | 0.18 | 2.17 | 49.19 | 11.90 |
| N16:0 | 709.584 | 22.1 | 92.36 | 205.62 | 53.70 |
| N17:0 | 723.599 | 0.34 | 2.51 | 87.76 | 13.59 |
| N18:1 | 735.599 | 0.05 | 0.58 | 9.61 | 3.14 |
| N18:0 | 737.615 | 2.58 | 11.31 | 275.47 | 43.69 |
| N19:0 | 751.631 | 0.04 | 0.31 | 83.70 | 9.91 |
| N20:1 | 763.631 | 0.08 | 0.52 | 18.29 | 1.31 |
| N20:0 | 765.646 | 0.39 | 1.93 | 64.50 | 9.11 |
| N21:0 | 779.662 | 0.15 | 0.93 | 18.48 | 1.52 |
| N22:1 | 791.662 | 0.45 | 14.14 | 13.86 | 0.24 |
| N22:0 | 793.677 | 0.89 | 9.48 | 11.96 | 1.44 |
| N23:1 | 805.677 | 0.02 | 1.90 | 3.28 | 0.04 |
| N23:0 | 807.693 | 0.39 | 2.53 | 1.89 | 0.28 |
| N24:2 | 817.677 | 2.88 | 22.22 | 1.36 | 0.21 |
| N24:1 | 819.693 | 5.27 | 79.17 | 2.64 | 0.43 |
| N24:0 | 821.709 | 1.04 | 4.91 | 1.27 | 0.26 |
| Total |  | 37.03 | 283.31 | 930.87 | 176.16 |

**Table S4 LPC species (nmol/g) in the lipids extracted from fresh fish bones and crustacean shells.**

| **Species** | **[LPC + Na]^+^** | **Grass carp bone** | **Black carp bone** | **Shrimp shell** | **Crab shell** |
| --- | --- | --- | --- | --- | --- |
| P16:0 | 502.327 | 0.60 | 0.26 | 1.05 | 0.74 |
| A16:0 | 504.342 | 3.92 | 0.85 | 1.77 | 1.78 |
| 16:0 | 518.322 | 10.38 | 2.61 | 2.91 | 1.81 |
| P18:0 | 530.358 | 0.88 | 0.37 | 0.39 | 0.42 |
| 18:2 | 542.322 | 4.17 | 2.52 | 1.41 | 0.58 |
| 18:1 | 544.337 | 10.78 | 4.19 | 1.73 | 2.41 |
| 18:0 | 546.353 | 3.19 | 0.75 | 1.89 | 0.61 |
| 20:4 | 566.322 | 8.95 | 1.01 | 0.11 | 0.21 |
| 20:3 | 568.337 | 3.17 | 0.37 | 0.04 | 0 |
| 22:6 | 590.322 | 2.90 | 0.37 | 0.12 | 1.18 |
| 22:5 | 592.337 | 7.78 | 0.56 | 0.04 | 0.06 |
| Total |  | 56.72 | 13.86 | 11.47 | 9.81 |

P, plasmenyl-; A, plasmanyl-.
